# Diet to Stop Hypertension: Should Fats be Included?

**DOI:** 10.1007/s11906-024-01310-7

**Published:** 2024-05-07

**Authors:** Paul J. Nestel, Trevor A. Mori

**Affiliations:** 1 Baker Heart and Diabetes Institute, Melbourne, Australia; 2 Medical School, Royal Perth Hospital Unit, University of Western Australia, Medical Research Foundation Building (M570), GPO Box X2213, Perth, WA 6847 Australia

**Keywords:** Diets, Dietary fats, Blood pressure, Hypertension, Food categories, Body weight, Population studies

## Abstract

**Purpose of Review:**

International guidelines emphasize advice to incorporate dietary measures for the prevention and in the management of hypertension. Current data show that modest reductions in weight can have an impact on blood pressure. Reducing salt and marine oils have also shown consistent benefit in reducing blood pressure. Whether other dietary constituents, in particular the amount and type of fat that play important roles in cardiovascular prevention, influence blood pressure sufficiently to be included in the management of hypertension is less certain. In this review, we provide a summary of the most recent findings, with a focus on dietary patterns, fats and other nutrients and their impact on blood pressure and hypertension.

**Recent Findings:**

Since reducing salt consumption is an established recommendation only corollary dietary advice is subject to the current review. Population studies that have included reliable evaluation of fat intake have indicated almost consistently blood pressure lowering with consumption of marine oils and fats. Results with vegetable oils are inconclusive. However dietary patterns that included total fat reduction and changes in the nature of vegetable fats/oils have suggested beneficial effects on blood pressure. Plant-based foods, dairy foods and yoghurt particularly, may also lower blood pressure irrespective of fat content.

**Summary:**

Total fat consumption is not directly associated with blood pressure except when it is part of a weight loss diet. Consumption of marine oils has mostly shown moderate blood pressure lowering and possibly greatest effect with docosahexaenoic acid-rich oil.

## Introduction

The search strategy includes articles published up to March 2024 derived from research involving human subjects, published in English and indexed in MEDLINE (through PubMed), EMBASE, the Cochrane Library, and other selected databases. Key search words included but were not limited to: “human”, “population studies”, “diets”, “blood pressure”, “hypertension”, “fats/oils”, “food categories”, “body weight”, “overweight/obesity”.

Hypertension is a major preventable risk factor for all-cause mortality and disability from cardiovascular diseases (CVD) [1, 2]. Hypertension is the main risk factor for death in Australia with approximately one in three adults having elevated systolic blood pressure (BP) [3]. Multiple risk factors contribute to this disorder of which overweight and obesity stand out. Other modifiable risk factors include lack of physical activity; an unhealthy diet, especially one high in sodium; excessive alcohol consumption; sleep apnea; high cholesterol; diabetes; smoking/tobacco use and stress. It has been estimated that 60–70% of hypertension in adults is attributable to adiposity [4]. Hypertension due to overweight and obesity impacts health in at least one-quarter of the world’s population [5]. The problem has been exacerbated by the global increased prevalence of obesity in recent decades; 10.8% of men and 14.9% of women were obese in 2014 [6]. By 2025, it is estimated that 18% of men and 21% of women globally will have obesity. The prevalence is even higher in the US with 42% of adults currently obese and anticipated to increase to 48.9% by 2030 [6]. It has been predicted that all American adults may be overweight or obese by 2048 [5]. Gaining excess weight has been shown to be a predictor of hypertension in normotensive individuals [7]. Data from the Framingham Study showed that compared with optimum BP, a normal BP at baseline associated with a 2-4 fold increased risk of hypertension, while high normal BP associated with a 5-12 fold raised odds. A 5% increase in weight over a 4-year interval associated with a 20–30% increased odds of being hypertensive. Conversely weight loss results in BP reduction although the relationship is not linear and weight regain is common [7, 8]. A 5% weight loss has been estimated to reduce systolic and diastolic BP by 3 mm Hg and 2 mm Hg among those with hypertension [6].

Clinical practice guidelines emphasize advice to incorporate dietary measures for the prevention and in the management of hypertension [9]. There is clear benefit in established measures such as reducing salt (NaCl) intake; population studies support such measures that lead also to reduced CVD [9]. A higher sodium-to-potassium (Na:K) ratio has been shown to be more strongly associated with BP outcomes than either sodium or potassium alone in hypertensive adult populations [10]. A meta-analysis of 85 trials showed that every 100 mmol/day reduction in urinary sodium excretion associated with lower mean systolic and diastolic BP by 5.56 mmHg (95% CI: − 4.52, − 6.59) and 2.33 mmHg (95% CI: − 1.66, − 3.00), respectively [11•]. A 100 mmol/d decrease in sodium intake associated with a reduction in mean systolic BP and diastolic BP of 7.79 mmHg (95% CI: − 4.90, − 10.67) and 3.10 mmHg (95% CI: − 1.37, − 4.83), respectively, in individuals with a baseline systolic BP < 140 mm Hg, and of 6.06 mmHg (95% CI: − 4.64, − 7.48) and 2.99 mmHg (95% CI: − 2.17, − 3.81) in participants with a baseline systolic BP ≥ 140 mm Hg.

Dietary sodium intake globally has not noticeably changed despite guidelines and much media publicity. Liu et al. recently reported assessment of sodium and potassium intake in 10,114 Chinese adults > 18 years as part of the China National Nutrition Survey 2015 [12•]. Using 24-h urine collections the estimated average daily sodium intake was ~ 4,400 mg/d, twice that recommended by the WHO upper limit of 2,000 mg/d, with 92% of adults exceeding the WHO upper limit. The average potassium intake was ~ 2,000 mg/d, < 60% of the WHO recommendation. Consequently, the mean Na:K ratio among Chinese adults was ~ 5, much higher than the public health guidance of < 1. Of note, the Na:K ratio was higher in those with hypertension than in those with normal BP. The findings by Liu et al. suggest an urgent need for policy changes in China to rectify the imbalance in dietary sodium and potassium intake, the successful implementation of which will likely avert a significant portion of BP related disease burden.

Numerous randomised controlled trials (RCTs) have shown potassium intake reduces BP. A systematic review and meta-analysis that included 25 RCTs involving 1,900 hypertensive subjects with mean age 24–75 years showed potassium supplementation decreased systolic BP by 4.48 mmHg (95% CI: 3.07, 5.90) and diastolic BP by 2.96 mmHg (95% CI: 1.10, 4.82) [13]. Therefore, the question has been asked as to whether salt-substitution, replacing a portion of NaCl with a potassium salt and thus decreasing the sodium content, represents a viable alternative at a population level. An open-label study in 20,995 Chinese participants among whom 88.4% were hypertensive was conducted in villages over 4.74 years comparing a salt substitute (75% sodium chloride and 25% potassium chloride) with their usual diet [14]. Salt substitution resulted in significantly fewer strokes and other cardiovascular events including death. In spite of these findings, it remains inconclusive as to whether a BP benefit would be achieved with population wide use of potassium-enriched salt substitutes [15]. The data are mixed on the BP-lowering effect of different potassium salts (potassium chloride, potassium citrate or potassium magnesium citrate) and there remains some concern whether the risk of hyperkalaemia might differ in at-risk patients.

Whether other dietary constituents, especially the amount and nature of fat that play key roles in CVD prevention also influence hypertension sufficiently to be included in the clinical management of hypertension is less certain with the probable exception of marine oil [16].

### Dietary Patterns to Reduce Blood Pressure

The”Dietary Approaches to Stop Hypertension” or DASH trial [17] has achieved almost gold-standard status against which other dietary measures are evaluated. It showed modest but clinically useful reductions in BP. The DASH diet focuses on increasing the intake of plant and lean meat/fish/dairy micronutrients associated with lowering BP (potassium, calcium, and magnesium) and limiting macronutrients and micronutrients associated with increased BP (saturated fat, added sugars, and sodium). A meta-analysis of 30 RCTs of DASH style diets [18], comprising 5,545 participants that included both normotensive and hypertensive individuals, showed in comparison with a control diet the DASH diet significantly reduced BP (systolic BP mean -3.2mmHg [95% CI, -4.2, -2.3 mmHg], diastolic BP mean -2.5 mmHg [-3.5, -1.5 mm Hg] both p < 0.001). Whilst hypertension status did not modify the effect on BP reduction, the effect was unsurprisingly greater with sodium reductions > 2400 mg daily than with < 2400 mg daily. Younger subjects (< 50 year) were more responsive than older subjects.

The dietary advice in a DASH diet includes changes beyond sodium reduction. The DASH diet emphasises low-fat or non-fat dairy products, lean meats, poultry, fish, a focus on vegetables, fruits, legumes, nuts, seeds, grains and vegetable oils, whilst limiting/avoiding saturated fat, fatty meats, refined grains, added sugars and alcohol. Collectively the DASH diet resembles dietary measures that also show benefit for CVD prevention as demonstrated in the long-term multicentre, longitudinal investigation of young Caucasian and African American adults of the CARDIA Study (Coronary Risk Development in Young Adults) [19]. A 30-year follow-up of the CARDIA Study showed participants following a more healthful dietary pattern associated with a 45% lower risk of type 2 diabetes (Hazard Ratio (HR) 0.55 [95% CI: 0.41, 0.74]) [20]. A healthful dietary pattern was determined using the validated A Priori Diet Quality Score (APDQS), which weighs 46 food groups rated as beneficial, neutral or adverse based on their known associations with CVD risk, and aligns with the 2015–2020 Dietary Guidelines for Americans [21]. A high APDQS dietary score associates with increased intake of fruits, vegetables, whole grains, low-fat dairy, fish, poultry, legumes, nuts, soy products, low-to-moderate alcohol, tea, and coffee, while limiting fast/fried foods, full-fat dairy, red meat, salty snacks, sweets, and sugar-sweetened soft drinks.

There is consensus that plant-based diets are associated with lower BP and overall better health outcomes including CVD when compared with animal-based diets [22•]. Plant-based diets provide many macro- and micronutrients including greater consumption of potassium and polyphenols, both of which have been shown to contribute to BP lowering [23, 24]. The CARDIA Study showed a dose-dependent relationship between plant food intake including (whole grains, refined grains, fruit, vegetables, nuts, or legumes) and reduced incidence of raised BP [19]. The relative hazards of elevated BP for quintiles 2–5 of plant food intake were 0.83 (95% CI: 0.68, 1.01), 0.83 (0.67, 1.02), 0.82 (0.65, 1.03) and 0.64 (0.53, 0.90), respectively, (P for trend = 0.01), compared with quintile 1.

Although the reduction in fat intake has been emphasized in the DASH diet, a comparison with a DASH-style diet but without restricting fat showed no advantage in lowering fat intake [25]. In a 3-period RCT, 36 healthy individuals (baseline mean systolic /diastolic BP of 134/85 mmHg) consumed in random order a control diet, a standard DASH diet, and a higher-fat, lower-carbohydrate modification of the DASH diet (HF-DASH diet) for 3 weeks each, separated by 2-wk washout periods. The HF-DASH diet was achieved by replacing non-fat and low-fat dairy with full-fat dairy products, thus increasing total and saturated fat, mostly whole milk, cheese and yogurt, and by reducing sugars, mostly from fruit juices. The HF-DASH diet lowered both systolic and diastolic BP to a similar extent as the DASH diet, suggesting the diet components responsible for the BP reduction were retained in the HF-DASH diet. However, the HF-DASH diet also reduced plasma triglycerides and VLDL concentrations without significantly increasing LDL cholesterol. Thus the long-term benefits of fat reduction remain less certain.

A feature of a number of dietary patterns that benefit BP, including the DASH diet, the Mediterranean diet [26] and the Nordic diet [27], is they all prioritise a diet high in fruit and vegetables, whole grains, legumes, seeds and nuts, fish, and dairy and low in meat and sweets and moderate alcohol intake. In a cross-sectional study of 328 overweight / obese participants (mean BMI 32.4kg/m^2^) evaluating six dietary patterns, a diet rich in nuts, seeds, fruit, and fish was inversely associated with BP [28]. A recent meta-analysis evaluated the effect on BP of seven plant-based dietary patterns comprising 41 clinical trials (8,416 participants of mean age 49.2 years) [29•]. In the pooled analysis, plant-based diets associated with lower systolic BP [DASH 5.53 mmHg (95% CI: 7.95,–3.12), Mediterranean 0.95 mmHg (–1.70,– 0.20), Vegan 1.30 mmHg (–3.90,1.29), lacto-ovo vegetarian 5.47 mmHg (–7.60,–3.34), Nordic 4.47 mmHg (–7.14,–1.81), high-fiber 0.65 mmHg (–1.83,0.53), high-fruit and vegetable 0.57 mmHg (–7.45,6.32)] and with similar effects on diastolic BP.

The Nordic [27] and Mediterranean [26] diets are geographic terms but are being used to describe unique patterns of food consumption. One feature of the Nordic Study is the consumption of berries that contain polyphenols that lower BP, as well as whole grains, rapeseed oil and nuts [27] whereas the Mediterranean diet is rich in olive oil, and includes legumes, whole grains, vegetables, and fruit, moderate intake of fish, low-to-moderate intake of dairy products (primarily as cheese and yogurt), moderate intake of wine, and low intake of red meat and processed meat [26]. An umbrella review of 50 systematic reviews and meta-analyses of RCTs [30•] compared 12 dietary patterns including DASH, Mediterranean, Nordic, vegetarian, low-salt, low-fat, portfolio (multiple candidate changes) and other single dietary component changes. The conclusion was that adherence to DASH, Nordic and portfolio diets were the most effective in lowering BP. The portfolio diet is a plant-based dietary pattern that combines recognized cholesterol-lowering foods including nuts, plant protein from soy products or dietary pulses (e.g. beans, peas, chickpeas, and lentils), viscous soluble fibre (e.g. oats, barley, psyllium, eggplant, apples, oranges, or berries) and plant sterols [31]. Among these dietary patterns, the DASH diet was associated with the greatest overall reduction in BP with unstandardized mean differences ranging from -3.20 mmHg (95% CI: -4.20, -2.30) to − 7.62 mmHg (95% CI: -9.95, -5.29) for systolic BP and from -2.50 mmHg (95% CI: -3.50, -1.50) to -4.22 mmHg (95% CI: -5.87, -2.57) for diastolic BP. A recent umbrella review of 341 meta-analyses of RCTs and 70 meta-analyses of observational studies has shown the DASH and Mediterranean dietary patterns, and restricting sodium intake with moderate alcohol intake, to be most effective for the management and prevention of hypertension [32••]. The DASH dietary pattern was the most effective for reducing systolic and diastolic BP; its effect was comparable to antihypertensive pharmacological treatment, with similar findings for the Mediterranean diet [32••].

The association between high quality plant-based foods, but not overall plant-based foods, and BP was reported from the INTERMAP Study (INTERnational study on MAcro/micronutrients and blood Pressure) from data in Japan, China, United Kingdom and United States [33]. A high-quality plant-based diet index was compared with an overall plant-based index and an unhealthy plant-based index. Blood pressures were significantly lower among those consuming a healthy plant-based index: for every 1 SD increase of the high-quality plant index, BP was reduced by -0.82 mmHg (95%CI: -1.32, -0.49) systolic BP and -0.49 mm Hg (95%CI: -0.91,-0.28) diastolic BP.

In addition to the aforementioned dietary patterns, a number of diet metrics have been developed to inform individuals and assess, compare and track risks of nutrient inadequacy and diet-related non-communicable diseases in populations. These include the Alternate Mediterranean Diet Score [34] that comprises vegetables, fruits, nuts, whole grains, legumes, fish, ratio of MUFA:SFA, red and processed meats and alcohol. The DASH diet quality score [35] is based on 9 nutrient targets (fat, saturated fat, protein, cholesterol, fibre, magnesium, calcium, sodium, and potassium) (score range, 0–9). The Alternative Healthy Eating Index-2010 (AHEI-2010) [36] includes foods and nutrients that associate consistently with lower risk of chronic disease and scored within a range from 0 (non-adherence) to 110 (perfect adherence). The Plant-Based Diet Index (PDI) includes a healthful PDI (hPDI) and unhealthful PDI (uPDI) and measures of adherence to a healthy and unhealthy plant-based diet, respectively [37, 38]. The Prime Diet Quality Score [39] includes 21 food groups, 14 of which are classified as healthy and 7 as unhealthy. Healthy food groups are assigned more points for higher consumption, with the scoring reversed for unhealthy groups. The Global Diet Quality Score (GDQS) [40] is a modification of the Prime Diet Quality Score and comprises 25 food groups with points assigned based on 3 or 4 categories of consumed amounts specific to each group. The score considers 16 healthy food groups (more points for higher intake), 7 unhealthy food groups (more points for lower intake), and 2 food groups classified as unhealthy when consumed in excessive amounts (points increase until specific amounts have been consumed after which no points are given). A recent report that included 12,002 men and women aged > 18 years from the 1997–2015 China Health and Nutrition Survey showed a higher GDQS associated with a lower risk of new-onset hypertension [41]. Multivariable adjusted relative risk (RR) of hypertension was 0.72 (95%CI: 0.62–0.83) for those with a high score (≥ 23) compared with individuals with a low GDQS score (< 15). Furthermore, a 25-percentile increase in the GDQS associated with a 30% (RR, 0.70; 95% CI, 0.64–0.76) lower risk of new-onset hypertension. Holzman et al. recently examined the Alternative Healthy Eating Index-2010 and DASH diet-quality in relation to BP in 677 women of age 25–55 years originally enrolled in the Pregnancy Outcomes and Community Health Study and followed up in the 7–15 years after delivery [42•]. Women with a poor diet-quality score (AHEI and DASH) had significantly higher mean systolic and diastolic BP.

A recent study investigated possible interactions between genotype and the DASH diet score, providing new insights into the mechanisms linking the DASH diet and BP [43]. The study examined interactions between the DASH diet score and over 9 million single nucleotide polymorphisms (SNPs) in six population groups (91% European) from the Cohorts for Heart and Aging Research in Genomic Epidemiology consortium (n = 35,660) and UK Biobank (n = 91,622). There were significant gene-diet interactions in 3 loci in European-specific analyses and 4 additional loci in cross-population analyses, suggesting that SNPs potentially interacting with the DASH diet may affect systolic BP via a DNA methylation-related mechanism. Studies with larger diverse populations will be required to validate these findings.

### Interventions with Individual Foods, Fats and Nutrients

The potential BP lowering through substituting types of fatty acids has found support in some studies. In the DIVAS Study (Dietary Intervention and VAScular function) 195 individuals were randomized to one of three trial arms each of 16-week duration providing isoenergetic diets that were rich in saturated fats, monounsaturated fats or n–6 polyunsaturated fats (PUFA) [44]. The group randomized to monounsaturated fatty acids substituting for saturated fatty acids achieved significant nocturnal systolic BP reduction (-4.9 mm Hg, p = 0.019) whereas BP in those receiving polyunsaturated fatty acid substitution failed to show benefit.

The most consistent effect on BP has been shown to occur when marine oils substituted for animal fat [16]. In one study significant BP lowering was achieved by the daily consumption of 3.65 g of omega-3 long chain polyunsaturated fatty acids especially when combined with weight loss of between 5 and 8 kg [45]. In overweight participants (mean BMI 31.6 kg/m2) following a 16-week dietary intervention, omega-3 long chain polyunsaturated fatty acids provided as dietary fish, and weight loss, had significant independent and additive effects on 24-h ambulatory BP. Effects were greatest on day-time systolic and diastolic BP (P = 0.01); relative to control with awake pressures -6.0/-3.0 mmHg with dietary fish alone, -5.5/-2.2 mm Hg with weight reduction alone, and -13.0/-9.3 mm Hg with fish and weight loss combined. These effects remained significant after adjustment for changes in urinary sodium, potassium, or the sodium/potassium ratio, as well as dietary macronutrients. Dietary fish also significantly reduced 24-h (-3.1 bpm) and awake (-4.2 bpm) ambulatory heart rates. The suggested effective daily dose of omega-3 fatty acids is > 2g and probably as much as 2–3 g as recently shown in a meta-analysis of 71 trials involving 4,973 individuals with a combined docosahexaenoic acid + eicosapentaenoic acid dose of 2.8 g/d (interquartile range, 1.3–3.6 g/d) [46•]. The analysis showed a non-linear association with BP with the optimal intake for systolic and diastolic BP reduction between 2 g/d (systolic BP, − 2.61 [95% CI: − 3.57, − 1.65]; diastolic BP, − 1.64 [95% CI: − 2.29, − 0.99]) and 3 g/d (systolic BP, − 2.61 [95% CI: − 3.52, − 1.69]; diastolic BP, − 1.80 [95% CI: − 2.38, − 1.23]). In subgroup analyses, there was a stronger and approximately linear dose–response relationship among hypertensive and hyperlipidaemic individuals, and in those > 45 years of age. Trials using larger numbers of subjects, longer duration and importantly higher dosage than the 1 g in the failed fish oil trials have shown more consistently beneficial cardiovascular outcomes [47].

Dietary fibre at least within fibre-rich foods appears to lower BP reasonably consistently. This effect has been demonstrated in both women and men. In the prospective cohort study in 28,926 female health professionals 8,722 became hypertensive over a 10-year period of annual observation [48]. After excluding known risk factors for BP, consumption of whole grains across quintiles associated inversely with risk for incident hypertension. The relative risks (RR) were 0.96 (95% CI: 0.89, 1.03), 0.95 (95% CI: 0.88, 1.02), 0.92 (95% CI: 0.85, 0.99), and 0.89 (95% CI: 0.82, 0.97) across the increasing quintiles of baseline whole-grain intake (P = 0.007 for the trend). Similar findings were found between whole-grain consumption among 51,529 men in the Health Professionals Follow-up Study: whole-grain consumption was less among the 9,227 men who became hypertensive over the 18 y of follow-up [49]. The relative risk of hypertension with increasing intake of grain was 0.81 (95% CI: 0.78, 0.92) highest versus lowest quintile of grain intake. It has been postulated that microbial activity which is associated with reduced BP may be one mechanism [50].

Dairy foods have been investigated for potential BP lowering effects. Studies included full-fat and reduced-fat products and fermented and unfermented dairy foods. In the CARDIA study the 15-year incidence of elevated BP was not related to dairy intake (in contrast to the positive association with consumption of meat) [19]. In a short-term double-blind randomized experimental study daily intake of 56 g whey protein lowered systolic BP by 3.9 mmHg and diastolic BP by 2.5 mmHg barely significantly (p = 0.05) compared with a control diet [51]. A report from the Framingham Heart Study Offspring Cohort provides information on specific dairy products [52]. During a median 14·6-year follow-up, 1,026 out of 2,340 participants developed incident hypertension. Consumption of total dairy foods and particularly low-fat and fat-free products including yogurt was associated with a modest reduction in the annualized increments in systolic BP and a lower risk of projected hypertension incidence; each additional serving of yogurt associated with a 6% (95%CI: 1, 10) reduced risk of incident hypertension.

Nutrition and dietary studies have shown increased food processing and more specifically ultra-processed foods, are associated with unhealthy outcomes, including CVD [53]. A recent umbrella review incorporating 14 eligible systematic reviews with meta-analyses identified high ultra-processed foods consumption is positively associated with obesity, diabetes, hypertension and mortality compared with low consumption [54•]. Current findings on the influence of ultra-processed foods on hypertension are inconsistent although a recent meta-analysis involving 111,594 participants showed higher consumption of ultra-processed foods associated with a 23% increased risk of hypertension in adults (95% CI: 1.11, 1.37; P = 0.034) [55]. The effect remained significant in subgroup analyses based on study design, sample size, geographical location, BMI, energy intake and physical activity.

Whether the source of dietary protein may influence the magnitude of the BP reduction following weight loss was studied in a controlled 16-week study in 36 post-menopausal women [56]. Equivalent and significant BP lowering was observed with a meat-based and an equicaloric plant-based diet with similar fat/oil intakes (meat consumers: (mean ± SD) 89.9 ± 14.4 kg baseline, 82.1 ± 13.5 kg end; plant consumers 80.2 ± 10.3 kg and 72.6 ± 9.2 kg respectively).

## Conclusions

**Fig. 1:**
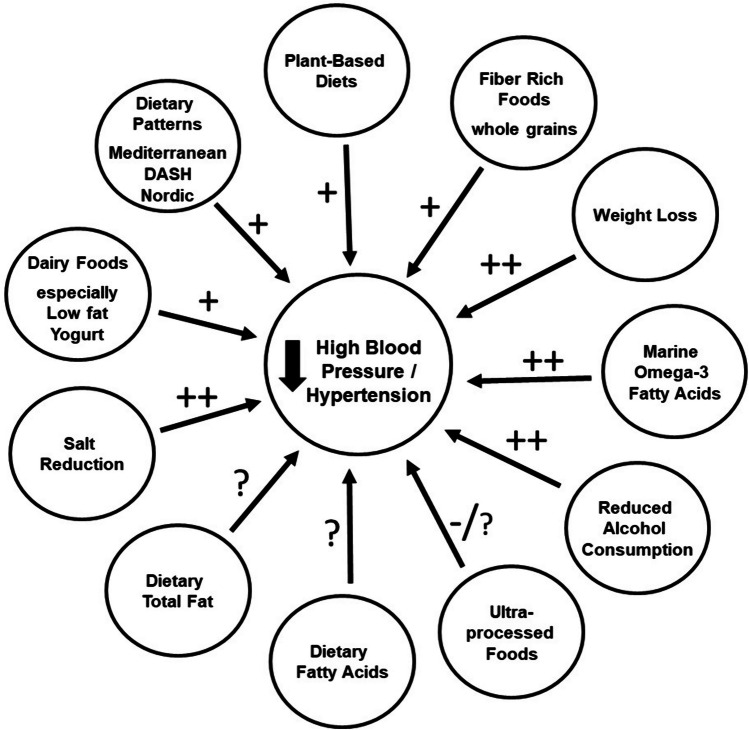
The effect of dietary patterns, fats, individual foods and nutrients in reducing high blood pressure / incidence of hypertension based on current evidence. + , beneficial effect; -, adverse effect; ?, uncertainty

Current evidence suggests the following with respect to dietary patterns, fats, individual foods and nutrients (Fig. 1):

1. Total fat consumption is not directly associated with BP except when it is part of a weight loss diet.
2. Individual fatty acids from vegetable oils do not appear to associate with BP.
3. Marine oils rich in long-chain polyunsaturated omega-3 fatty acids, particularly docosahexaenoic acid, are more likely to associate with lower BP than other edible oils or fats.
4. Patterns of eating that emphasize plant foods appear to associate with lower BP.
5. Higher consumption of specific foods including fibre-rich grains and yogurt has shown reasonably consistent association with lower BP.
6. Populations in regions around the Mediterranean and possibly in Nordic parts of Europe with their characteristic patterns of eating appear to have lower mean BP than in other western European countries.

## Data Availability

No datasets were generated or analysed during the current study.
